# Ibrutinib for chronic lymphocytic leukemia in the setting of respiratory failure from severe COVID‐19 infection: Case report and literature review

**DOI:** 10.1002/jha2.98

**Published:** 2020-09-20

**Authors:** Adam Yuh Lin, Michael J. Cuttica, Michael G. Ison, Leo I. Gordon

**Affiliations:** ^1^ Division of Hematology/Oncology, Department of Medicine Northwestern University Feinberg School of Medicine and the Robert H. Lurie Comprehensive Cancer Center Chicago Illinois; ^2^ Division of Pulmonary and Critical Care, Department of Medicine Northwestern University Feinberg School of Medicine Chicago Illinois; ^3^ Division of Infectious Diseases, Department of Medicine Northwestern University Feinberg School of Medicine Chicago Illinois

**Keywords:** acute respiratory distress syndrome, BTK inhibitor, COVID‐19, chronic lymphocytic leukemia, Ibrutinib

## Abstract

Ibrutinib, a known Burton's tyrosine kinase (BTK) and interleukin‐2 inducible T‐cell kinase (ITK) inhibitor, is used for the treatment of B‐cell disorders (chronic lymphocytic leukemia [CLL] and various other lymphomas) and chronic graft versus host disease following allogeneic hematopoietic cell transplantation. Because it is considered an immunosuppressant, continuation of ibrutinib is often debated when patients have an active infection, and this becomes an especially difficult decision in the setting of coronavirus disease 2019 (COVID‐19). Here, we describe a patient with CLL who was on ibrutinib then developed severe COVID‐19 infection requiring mechanical ventilation. We elected to continue ibrutinib the same day he was intubated, reasoning that BTK inhibition in myeloid immune cells has been shown to reduce or even reverse influenza‐mediated acute lung injury and that ITK inhibition in T cells has correlated with reduction in viral replication, and therefore may have an advantage in this setting. Ibrutinib also has been shown to block Src family kinases, which potentially could result in reduction of viral entry and the inflammatory cytokine response in the lungs. The patient was extubated after 9 days with a complex hospital course and eventually discharged on room air. The only way to rationally inform these decisions and explore similar potentially promising leads in this pandemic is to conduct carefully done clinical trials.

## 1 INTRODUCTION

Incidence and mortality due to severe acute respiratory syndrome coronavirus 2 (SARS‐CoV‐2), which causes coronavirus disease 2019 (COVID‐19), continue to rise globally. Known risk factors for increased disease severity and mortality include older age, co‐morbidities, and immune suppression [1]. Cancer patients, especially those with hematologic disorders, are often on treatment regimens that suppress the immune system, and have been shown to have worse outcomes when infected with SARS‐CoV‐2 [2]. Questions about continuation of these agents during COVID‐19 is heavily debated.

Ibrutinib is an extremely potent inhibitor of Burton's tyrosine kinase (BTK), which is important for B‐cell growth and proliferation. Therefore, ibrutinib has been approved by the Food and Drug Administration (FDA) for use in chronic lymphocytic leukemia (CLL), mantle cell lymphoma, marginal zone lymphoma, and chronic graft versus host disease (GvHD). In the ILLUMINATE study, which led to FDA approval of ibrutinib as front‐line therapy for CLL, ibrutinib decreased inflammatory cytokines associated with infusion‐related reactions when patients received the anti‐CD20 antibody obinutuzumab [3]. Ibrutinib's immune suppressive activity was the basis of approval for chronic GvHD in allogeneic hematopoietic cell transplant patients (overall response rate 67%) [4]. However, there is concern that ibrutinib could exacerbate infection in susceptible patients. Treon et al described six patients with Waldenstrom macroglobulinemia who were on ibrutinib and diagnosed with COVID, and found that only one out of six patients required hospitalization [5]. Here, we describe a patient with CLL on ibrutinib, who developed severe COVID‐19 infection, and we discuss the scientific rationale that informed our decision‐making process at the bedside.

## 2 CASE DESCRIPTION

Our patient is a 77‐year‐old male with CLL, 13q del diagnosed in 2005 and was started on ibrutinib 420 mg/day in March 2016 for progressive disease. He has been stable for 4 years. He has required intravenous immunoglobulin infusions for hypogammaglobulinemia periodically every 1–2 months during the winter months. On March 11, 2020, he presented to an urgent care facility with 4 days of sore throat, fevers up to 101°F, slight dry cough after returning from an overseas trip. He denied shortness of breath and had SpO_2_ of 96%. Point of care influenza test was negative and strep antigen swab was positive, and therefore COVID‐19 testing was not sent.

Six days later (March 17, 2020), he presented to the emergency room with progressive symptoms and oxygen saturation dropped to 89% on 2L nasal canula (NC). Nasal swab COVID‐19 testing was positive. He was admitted to the COVID‐19 medical intensive care unit (MICU) due to further increased oxygen demand. Ibrutinib was stopped on admission. However, it was unclear if the patient was taking it the last few days when he was feeling unwell.

On the second day of admission, he was intubated for progressive acute hypoxemic respiratory failure, which was about 10 days after his first reported symptoms. From the data reported from Wuhan, the median onset of illness to invasive mechanical ventilation was 14.5 days (range 12–19 days) [1]. With his older age and underlying CLL, there was concern that his clinical course would be more aggressive. Ventilation was set for lung protective strategy targeting a tidal volume of 6 mL/kg ideal body weight with high positive end expiratory pressure, coupled with aggressive airway clearance.

**FIGURE 1:**
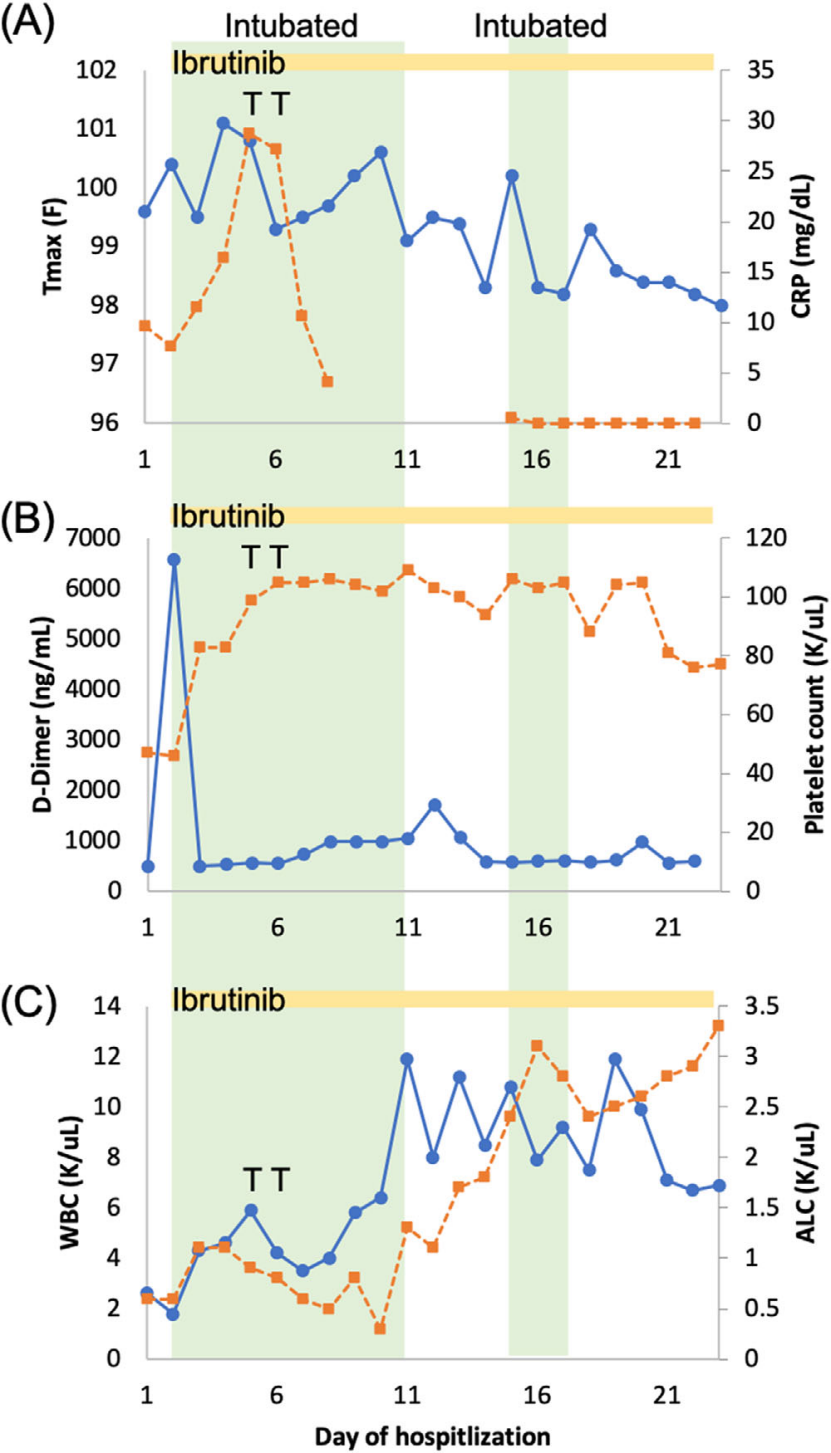
Laboratory data of CLL patient on ibrutinib with severe COVID‐19 infection. A, Maximum temperature in 24 hours (*T*_max_) (blue) and C‐reactive protein (orange). B, D‐dimer (blue) and platelet count (orange). C, White blood cell count (WBC) (blue) and absolute lymphocyte count (ALC) (orange) in relation to day of hospitalization. Green shaded areas: intubated. Yellow bar: ibrutinib use. T: tocilizumab

After an interdisciplinary discussion, ibrutinib was resumed at a higher dose of 560 mg (starting day 2 of admission). Although inhibition of BTK with ibrutinib is covalent, inhibition of other immune‐mediated kinases is noncovalent, and thus higher dose were used to achieve enough inhibition [6]. He also received one dose of hydroxychloroquine on admission, which was not continued, and two doses of tocilizumab on days 5 and 6 for recurring fever and increasing C‐reactive protein (CRP). His fever resolved and CRP improved after tocilizumab (Figure 1A). D‐dimer levels initially increased from 484 ng/mL on admission to 6573 ng/mL, and declined after starting ibrutinib (Figure 1B). Similarly, the platelet count that was as low as 47 K/μL on admission improved to >100 K/μL. He also had normalization of leukopenia and lymphopenia after ibrutinib (Figure 1C).

On day 11 (March 27, 2020), he was extubated to high flow nasal canula (HFNC) and transferred to a COVID unit on day 14 of admission (March 30, 2020) on 2L NC. Unfortunately, he developed recurrent acute hypoxemic respiratory failure the following night (March 31, 2020) and was found to have a large mucous plug in his left mainstem bronchus during reintubation and BAL. He developed leukocytosis and BAL showed Gram‐positive cocci, suggesting a new ventilator‐associated pneumonia (VAP). SARS‐CoV‐2 PCR remained positive but the clinical deterioration was thought to be due to the VAP and not due to COVID‐19. He was extubated 2 days later on day 17 of admission to HFNC (April 2, 2020), and was transferred to the floor. He was weaned off oxygen 3 days later and discharged to an acute inpatient rehabilitation on April 14, 2020 (day 28). On discharge, he was switched from ibrutinib 560 mg to his previous 420 mg dosing. After 2 weeks of rehab, the patient went home and has returned to his prior normal state of health.

## 3 DISCUSSION AND LITERATURE REVIEW

This patient presented a difficult choice in management that is now confronting clinicians and investigators who manage patients with underlying malignancy and COVID‐19 around the world. Since the pandemic, several articles have been published regarding CLL and COVID‐19. Surveying 62 CLL specialists, Koffman et al found that only 44% of experts favored unconditional continuation of treatment for COVID‐19 patients in the outpatient setting and down to 32.5% in non‐ICU inpatients [7]. Mato et al noted that patient with CLL who were receiving BTK inhibitors and diagnosed with COVID‐19 had similar survivals (34%) compared with patients who did not receive BTK inhibitors (35%); however, 79% of patients had their BTK inhibitor held at the time of symptomatic COVID‐19 diagnosis [8]. Most of the discontinuation decisions were due to concerns of the increased infections risks with BTK inhibitor use. On the other hand, hospitalization rate for severe COVID‐19 infections was significantly lower for CLL patients who were on ibrutinib prior to diagnosis compared with those on other CLL‐directed therapies or off therapy [9].

Our reasoning for continuing the ibrutinib in this patient was based on data that suggested that there might be reduction in viral replication by IL‐2 inducible kinase (ITK) inhibition and that BTK inhibition might reduce acute lung injury. A key feature of patients with severe, progressive COVID‐19 appears to be a cytokine storm that contributes to a series of complications, including lung injury, DIC, and myocardial complications. Similar to COVID‐19 infections, autopsy specimens of SARS‐CoV‐1 patients showed that angiotensin converting enzyme 2 (ACE2) positive cells had increased expression of tumor necrosis factor (TNF)‐α,  IL‐1β, IL‐6, C‐X‐C motif chemokine 10 (CXCL10) or interferon γ‐induced protein 10 kDa (CXCL10/IP10), and monocyte chemoattractant protein‐1 (MCP‐1) that appeared causally related to the acute lung injury and pathogenesis observed [10]. Therefore, selective immunosuppression may modulate this response and improve outcomes.

Roschewski et al described 19 patients with COVID‐19 that was given another BTK inhibitor, acalabrutinib, off label [11]. They found that overall 10 out of the 19 patients who were hospitalized and needed supplemental oxygen or intubation were successfully discharged on room air. These patients had improved lymphocyte count similar to our patient after starting acalabrutinib. However, these patients were not previously on BTK inhibitors and did not have an underlying hematologic malignancy.

*BTK inhibition reduces lung injury*. In addition to expression on B cells, BTK is expressed in myeloid immune cells including macrophages, monocytes, and neutrophils [12]. BTK binds and activates the toll‐like receptor 4 (TLR4) pathway in these cells [13, 14], which leads to induction of proinflammatory cytokines by the NFkB pathway [15]. Silencing BTK by intratracheal delivery of BTK siRNA caused suppression in NFkB, p38 MARK, and iNOS signaling pathways, which resulted in protection of acute lung injury (ALI) and acute respiratory distress syndrome (ARDS) in a murine sepsis model [16]. Mice that received BTK siRNA showed less interstitial lung edema and inflammatory cell infiltration 12 hours after establishment of bacterial sepsis. Isolated alveolar macrophages had reduced TNF‐α, IL‐1β, and IL‐6 secretion when stimulated by lipopolysaccharides (LPSs), a bacteria‐derived TLR4 agonist. Similarly, Krupa et al showed that BTK blockade in alveolar neutrophils reduced lung edema, alveolar septa thickening, and inflammatory infiltration compared with untreated mice in an ALI model [17]. Ibrutinib inhibition of BTK has also been shown to reduce myeloid cell inflammatory response during pneumococcal pneumonia induced ALI [18].

Furthermore, inhibition of BTK by ibrutinib rescued mice from lethal influenza‐induced ALI [19]. In an influenza A virus‐associated lung injury model, all mice treated with saline died or lost more than 30% of body weight, while ibrutinib‐treated mice had a weight loss nadir at day 4 and recovered to normal by day 8–9. The ibrutinib‐treated mice had less inflammatory cell recruitment on histology and reduced appearance of ground glass opacities on CT. These mice also showed less total protein and WBC in BAL fluid. Taken together, these results support the notion that BTK inhibition maybe a new drug target for viral‐induced ARDS.

*ITK inhibition reduces viral replication*. ITK activation causes T‐cell activation, cytokine release, and rapid proliferation [20]. T‐cell‐mediated immunity plays a role in clearance of viral infections [21]. ITK is upregulated on T‐cell membranes after infection with influenza. Importantly, g shRNA that targets and downregulates ITK leads to inhibition of influenza replication in infected T cells. Ibrutinib is an irreversible inhibitor of ITK and inhibits the formation of Th2 immunity, while Th1 CD4 T cell and CD8 T cells remain mostly active due to other redundant tyrosine kinases [22]. In CLL patients, as in our patient above, ibrutinib may maintain interferon‐ γ production from Th1 cells but with a reduction in IL‐4 secreting Th2 T cells in peripheral blood. Furthermore, ibrutinib was able to rescue antigen‐specific CD8 T‐cell response in an immune suppressive leukemia mouse model (EuTCL1). CD8 T cells in CLL patients on chronic ibrutinib had less PD1 expression, allowing them to be more active [23]. In addition, up to 74% of CLL patients on ibrutinib were able to achieve seroprotective titer antibody response to influenza after receiving the vaccine [24]. These results suggest that ITK blockade with ibrutinib can reduce viral replication while maintaining important Th1 immunity to synergistically lead to elimination of the virus.

Ibrutinib also targets other kinases, which may contribute to better outcomes in viral infections. Src family kinase (SFK) inhibition blocks single‐stranded RNA virion assembly and entry. Ibrutinib is known to inhibit HCK, part of the SRC family of kinases, which is expressed in lung tissue. Targeting of SFK with saracatinib led to inhibition of Middle East respiratory syndrome coronavirus (MERS‐CoV) replication [25]. HCK regulates ADAM17 [26], which can cleave ACE2, freeing SARS‐CoV‐2 to bind to ACE2 receptors. It is reasonable to hypothesize then that ibrutinib can minimize SARS‐CoV‐2 replication and entry by inhibition of SFK and HCK. In addition, HCK activation in macrophages leads to extensive pulmonary inflammation, and therefore, inhibition of HCK could minimize ALI [27].

*Ibrutinib may be harmful in the setting of active infection*. Concerns of ibrutinib worsening infections are valid. The immunosuppressive activity of ibrutinib has been associated with an increased incidence of invasive aspergillosis. This may be associated with BTK involvement in the TLR‐9 endosomal associated response to aspergillosis in macrophages, which is critical to clearance of this fungus [28]. There is also an increase in atypical *Pneumocystis jirovecii* infections with patients on ibrutinib and use of prophylactic antibiotics has been recommended by some [29]. Furthermore, there is some evidence that ibrutinib can worsen bleomycin‐associated pulmonary fibrosis [30]. However, Cubillos‐Zapata et al found that during the first cycle of ibrutinib therapy, phospho‐ERK1/2 protein levels and antigen presentation in CD14^+^ cells, which are mainly macrophages, increased to normal levels when stimulated with bacterial stimuli (LPS). These CD14+ cells could provide some bacterial infection protection for CLL patients [31].

## 4 CONCLUSION

By targeting BTK, ITK, and others kinases and intermediates, ibrutinib has the potential to minimize inflammation and prevent ALI or ARDS and to reduce viral replication. In our case, the patient was restarted on ibrutinib and had improvement in D‐dimer levels and absolute lymphocyte count fairly quickly. However, it is important to note that due to the use of tocilizumab and other medications, it is difficult to link his apparent recovery to directly ibrutinib. Although logically BTK inhibitors should have efficacy in preventing or treating ALI in COVID‐19, further studies in animal models as well as clinical trials are required to define the optimal role of ibrutinib in patients with life‐threatening viral infections.

## CONFLICT OF INTEREST

The authors declare no conflict of interest.

## AUTHOR CONTRIBUTIONS

Adam Yuh Lin and Leo I. Gordon were the main authors of the manuscript. Michael J. Cuttica and Michael G. Ison contributed to editing and scientific discussion of the manuscript. Leo I. Gordon, Michael J. Cuttica, and Michael G. Ison provided care to the patient described in the article.
